# Cysteinyl-leukotrienes promote cutaneous Leishmaniasis control

**DOI:** 10.3389/fcimb.2023.1192800

**Published:** 2023-06-12

**Authors:** Letícia Paula Trajano Noronha, Monique Daiane Andrade Martins, Archimedes Barbosa Castro-Junior, Maria Luiza Thorstenberg, Laís Costa-Soares, Thuany Prado Rangel, Felipe Carvalho-Gondim, Bartira Rossi-Bergmann, Luiz Eduardo Baggio Savio, Claudio de Azevedo Canetti, Robson Coutinho-Silva

**Affiliations:** ^1^ Laboratory of Immunophysiology, Instituto de Biofísica Carlos Chagas Filho, Universidade Federal do Rio de Janeiro, Rio de Janeiro, Brazil; ^2^ Laboratory of Immunopharmacology, Instituto de Biofísica Carlos Chagas Filho, Universidade Federal do Rio de Janeiro, Rio de Janeiro, Brazil; ^3^ Laboratory of Inflammation, Instituto de Biofísica Carlos Chagas Filho, Universidade Federal do Rio de Janeiro, Rio de Janeiro, Brazil

**Keywords:** *Leishmania amazonensis*, cysteinyl-leukotrienes, cutaneous leishmaniasis (CL), LTC_4_, LTD_4_, P2X7

## Abstract

Leishmaniasis is a neglected tropical parasitic disease with few approved medications. Cutaneous leishmaniasis (CL) is the most frequent form, responsible for 0.7 - 1.0 million new cases annually worldwide. Leukotrienes are lipid mediators of inflammation produced in response to cell damage or infection. They are subdivided into leukotriene B4 (LTB_4_) and cysteinyl leukotrienes LTC4 and LTD4 (Cys-LTs), depending on the enzyme responsible for their production. Recently, we showed that LTB_4_ could be a target for purinergic signaling controlling *Leishmania amazonensis* infection; however, the importance of Cys-LTs in the resolution of infection remained unknown. Mice infected with *L. amazonensis* are a model of CL infection and drug screening. We found that Cys-LTs control *L. amazonensis* infection in susceptible (BALB/c) and resistant (C57BL/6) mouse strains. *In vitro*, Cys-LTs significantly diminished the *L. amazonensis* infection index in peritoneal macrophages of BALB/c and C57BL/6 mice. *In vivo*, intralesional treatment with Cys-LTs reduced the lesion size and parasite loads in the infected footpads of C57BL/6 mice. The anti-leishmanial role of Cys-LTs depended on the purinergic P2X7 receptor, as infected cells lacking the receptor did not produce Cys-LTs in response to ATP. These findings suggest the therapeutic potential of LTB4 and Cys-LTs for CL treatment.

## 1 Introduction

Leishmaniasis is a neglected tropical parasitic disease affecting more than 12 million people worldwide, with 0.7–1.0 million new cases yearly (Burza et al., 2018). *Leishmania* parasites come in two forms: promastigotes, which are flagellated and extracellular, and amastigotes, intracellular and with a retracted flagellum. Leishmania promastigotes enter hosts through the bite of sand fly vectors and infect macrophages, where they differentiate into amastigotes. Depending on the parasite species and the patient’s immunological status, leishmaniasis can manifest as tegumentary leishmaniasis or visceral leishmaniasis. Tegumentary leishmaniasis can present as cutaneous leishmaniasis (CL), characterized by one or more high-edged wounds (Martins et al., 2014), mucosal leishmaniasis, characterized by mutilations in the oropharyngeal mucosa due to activation of the immune system and low parasitic load, diffuse cutaneous leishmaniasis, with non-ulcerated lesions spread across the skin, resulting from an anergic immune response and high parasitic load, and disseminated CL, caused by the spread of the parasite from the cutaneous lesion through the blood or lymph (Desjeux, 2004).

CL is the most common form, accounting for 90% of cases; all dermotropic species may cause it. After inoculation by sandflies, flagellated promastigotes infect skin phagocytes (neutrophils and macrophages), transforming and multiplying as amastigotes within phagolysosomes. Skin lesions develop slowly through macrophage infection and are often restricted to the bite site. Initially, a nodule forms and progresses chronically, eventually ulcerating as the inflammatory reaction intensifies. Many microbicidal intracellular innate mechanisms control infection by *Leishmania*, and macrophages are among the primary immune cells involved in parasite elimination through the degradation of phagolysosomal enzymes, the release of cytokines, and the production of reactive oxygen species and nitric oxide. The latter is the most critical control mechanism against phagocytosed intracellular microorganisms such as *Leishmania* (Moradin and Descoteaux, 2012).

During infection, nucleotides (well-recognized danger signals) are released into the extracellular environment and act by activating purinergic receptors under various pathological conditions (Virgilio et al., 2020). ATP nucleotides activate the P2X7 and P2Y_2_ receptors and control *L. amazonensis* infection via leukotriene (LT) B_4_ production (Chaves et al., 2014a; Chaves et al., 2016; Thorstenberg et al., 2018; Chaves et al., 2019). The first step for LTs synthesis is the release of arachidonic acid (AA) from membrane phospholipids hydrolyzed by the phospholipase A2. AA is oxygenated by 5-lipoxygenase (5-LO), which, with 5-LO activating protein, forms the unstable precursors of all other LTs, named LTA_4_ (Murphy and Gijón, 2007). LTA_4_ is converted by LTA_4_ hydrolase to LTB_4_, or it can be conjugated with reduced glutathione by LTC_4_ synthase to yield LTC_4_. LTB_4_ and LTC_4_ are exported to extracellular space by specific transporter proteins. The released LTC_4_ is converted to LTD_4_ and then to LTE_4_ by sequential amino acid hydrolysis.

LTB_4_ is an activator and chemotactic agent for leukocytes involved in pathophysiological conditions such as asthma and atherosclerosis. LTB_4_ is also essential in controlling *L. amazonensis* infection through augmented nitric oxide production in infected macrophages (Serezani et al., 2006). Cys-LTs (LTC_4_, LTD_4_, and LTE_4_) participate in the pathogenesis of allergic asthma inflammation by recruiting leukocytes and enhancing vascular permeability (Capra et al., 2007). However, the role of Cys-LTs in parasite infection is poorly understood. According to the literature, LTC_4_ enhances the association of mouse peritoneal macrophages with *Trypanosoma cruzi*, increasing its uptake and intracellular destruction (Wirth and Kierszenbaum, 1985).

Leishmaniasis control in endemic regions is based on chemotherapy and the management of reservoirs or vectors. Chemotherapy has several shortcomings in terms of toxicity, development of resistance, stability, and cost. Furthermore, they are long-term treatments, have low tolerability, and are poorly adapted to remote areas, making them challenging to administer because their administration usually requires a hospital environment (DNDi Annual Report, 2016). These limitations drove researchers worldwide to identify new medications for leishmaniasis.

In this study, we assessed the role of Cys-LTs in controlling *L. amazonensis* infection in macrophages isolated from susceptible (BALB/c) and resistant (C57BL/6) mouse strains. We analyzed the *in vitro* effect of Cys-LTs on infected peritoneal macrophages and *in vivo* intralesional treatment in infected mouse footpads. We showed that Cys-LTs diminished the infection index in cultured macrophages and reduced lesion size and parasite load in footpads.

## 2 Materials and methods

### 2.1 Chemicals

ATP was purchased from Sigma-Aldrich (St. Louis, MO, USA). LTB_4_, LTC_4_, and LTD_4_ were obtained from Cayman Chemical (Ann Arbor, MI, USA).

### 2.2 Animals

We used male and female 8–10-week-old BALB/c, wild-type C57BL/6, and P2X7 knockout mice (P2X7^-/-^; Jackson Laboratory, USA). The experiments, maintenance, and care of mice were carried out according to the Brazilian College of Animal Experimentation guidelines. The mice were housed in a temperature-controlled room (26°C) with a light/dark cycle (12 h). Food and water were provided *ad libitum*. For *ex vivo* experiments, mice were anesthetized in a carbon dioxide (CO_2_) chamber and sacrificed by cervical dislocation. The Ethics Committee on the Use of Animals (CEUA) approved all experimental protocols (IBCCF, UFRJ n° 077/15 and n° 152/21).

### 2.3 Parasites

The *L. amazonensis* (MHOM/BR/Josefa strain) was used for *in vitro* and *in vivo* experiments. Amastigotes isolated from mouse lesions (from BALB/c mice) were allowed to transform into axenic promastigotes forms by growth at 24°C for 7 days in 199 medium (Sigma-Aldrich, St. Louis, MO) supplemented with 10% heat-inactivated fetal bovine serum (FBS; Gibco, Thermo Fisher Scientific, MA), 2% male human urine, 1% L-glutamine, and 0.25% hemin. Promastigotes in the late stationary growth phase were used until the tenth passage to preserve parasite virulence.

### 2.4 Macrophage isolation, cell culture, and infection

Peritoneal macrophages were harvested from the peritoneal cavity by washes with cold phosphate-buffered saline (PBS). Cells were directly seeded on culture plates in RPMI 1640 medium, at 37°C, with 5% CO_2_. After 1 h, cultures were washed gently with PBS (twice) to remove non-adherent cells. The cells were cultured for 24 h in RPMI 1640 supplemented medium (10% FBS and 100 units penicillin/streptomycin and 2 mM L-glutamine) at 37°C and 5% CO_2_. Then cells were infected for 4 h with *L. amazonensis* promastigotes (MOI 10:1 - *Leishmania*:macrophage) at 37°C. The non-internalized parasites were removed by extensive washing with sterile PBS at 37°C. Then, infected cells were maintained in an incubator at 37°C and 5% CO_2_ until stimulation.

### 2.5 Pharmacological treatments

Infected macrophages were treated with 100 nM LTB_4_, LTC_4,_ or LTD_4_ for 30 minutes at 37°C and 5% CO_2_. Then, cell monolayers were washed with PBS and maintained in RPMI 1640 supplemented at 37°C and 5% CO_2_ for 24 h.

### 2.6 Macrophages infection index

Intracellular parasite loads were analyzed as previously described (Thorstenberg et al., 2018). Briefly, mouse peritoneal cells were infected, treated with nucleotides, fixed onto slides, and stained with a panoptic stain (Laborclin ^®^, PR, Brazil). Parasites were counted using a Primo Star light microscope (Zeiss, Germany) with a 40X objective (100X for representative pictures). Images were acquired using a Bx51 camera (Olympus, Tokyo, Japan) using CellF software. We calculated the “infection index” representing the overall infection load, based on 100 cells in five fields to obtain the number of infected macrophages and the average number of parasites per macrophage. Individual amastigotes were visible in the cytoplasm of infected macrophages. The results were expressed as infection index II = (% infected macrophages) × (amastigotes/infected macrophages)/100.

### 2.7 *In vivo* infection and pharmacological treatments

Mice were infected in the footpad by intradermal injection of 10^6^ *L. amazonensis* promastigotes in PBS. Intralesional treatment with 20 µL of 5 ng LTD_4_ or vehicle for 3 weeks, twice a week, started 7 days post-infection (dpi). Lesion growth was calculated by evaluation of the “swelling” (i.e., the thickness of the infected footpad – thickness of the uninfected footpad from the same mouse) using a traditional caliper (Mitutoyo^®^). Forty-eight hours after the final injection (26 dpi), animals were euthanized. For parasitic load determination, the infected footpad and popliteal lymph nodes were removed and dissociated (in M199 supplemented culture medium).

### 2.8 Parasite load determination

The parasite load in the mice-infected tissues was determined using a limiting dilution assay, as previously described (Titus et al., 1985; Thorstenberg et al., 2018). Mice were euthanized in a CO_2_ chamber, followed by cervical dislocation. Footpads and lymph nodes were collected and weighed, and cells from the whole footpad and draining lymph nodes were dissociated using a 40-μm cell strainer (BD^®^) in PBS. Large pieces of tissue debris were removed by centrifugation at 150 x *g*. The cells were then separated by centrifugation at 2,000 × *g* for 10 min and resuspended in supplemented M199. The samples were cultured in 96-well flat-bottom microtiter plates (BD^®^, USA) at 26–28°C. After a minimum of 7 days, wells were examined using phase-contrast microscopy in an inverted microscope (NIKON TMS, JP) and scored as “positive” or “negative” for the presence of parasites. Wells were scored “positive” when at least one parasite was observed per well.

### 2.9 Cys-LTs determination assay

Peritoneal macrophages (2 x 10^5^ per well) from C57BL/6 and P2X7^-/-^ mice were plated in 96-well plates in triplicate and infected or left untreated after twenty-four hours. At the end of the incubation period, the supernatants were removed, and a new medium with 5% FBS was added. The cultures were then treated with ATP for 30 min with subsequentially medium change. The culture supernatants were collected 30 min and 3 h after ATP treatment and stored at −80°C until analysis. To measure the Cys-LTs released in the supernatant of cells, enzyme immunoassays were performed using a Cys-LTs enzyme immunoassay kit (Cayman Chemical™) following the manufacturer’s instructions. The cysteinyl leukotriene enzyme immunoassay kit does not distinguish among Cys-LTs. The cross-reactivity of the assay is 100% for LTC4 and LTD4 and 79% for LTE4. The cross-reactivity to LTB4 is 1.3% and 0.04% to 5(S)-HETE. The overall amount of LTC_4_, LTD_4,_ and LTE_4_ was measured in each sample.

### 2.10 Statistical analysis

Statistical analysis were performed using Student’s t-test when comparing two groups. For more than two groups, data were analyzed using a one-way analysis of variance followed by Tukey’s multiple comparison *post hoc* test using Prism 8.0 software (GraphPad Software, La Jolla, CA). Differences between the experimental groups were considered statistically significant at *P <*0.05.

## 3 Results

### 3.1 Cys-LTs (LTC_4_ and LTD_4_) decreased the parasite load of *L. amazonensis* in peritoneal macrophages from BALB/c and C57BL/6 mice

**Figure 1:**
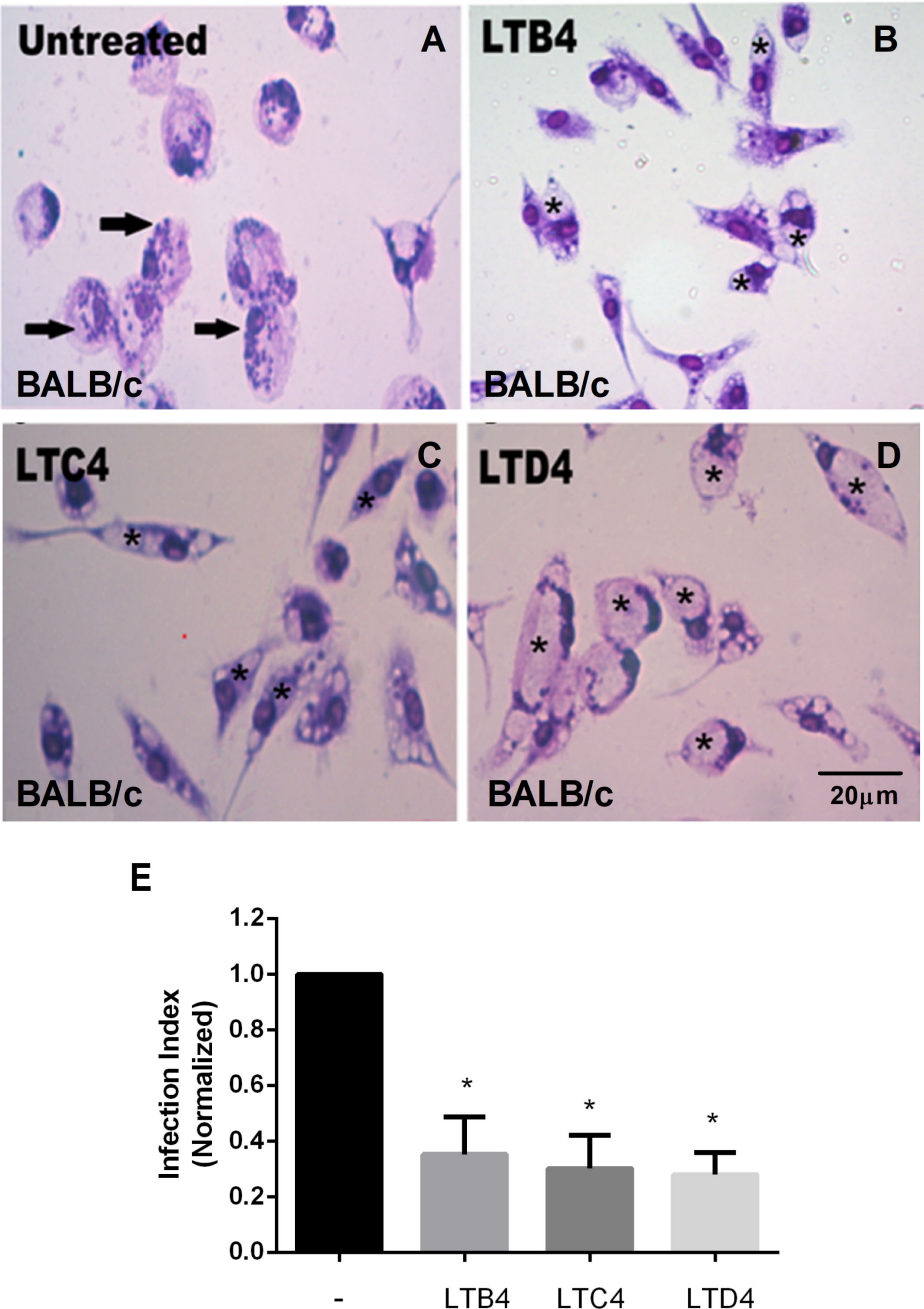
LTs reduce the parasite burden in infected BALB/c mouse macrophages. Peritoneal macrophages from BALB/c mice were infected with *L. amazonensis* promastigotes at 10:1 (*Leishmania*:macrophage). After 4 h, the free parasites were washed, and after 24 h, infected cells were treated **(B–D)** or not **(A)** with 100 nM of LTB4 **(B)**, cysteinyl leukotrienes LTC_4_ **(C)**, and LTD_4_ **(D)**. Twenty-four hours later, cells were stained with May Grunwald-Giemsa, and the infection index was determined by direct counting under light microscopy. Arrows indicate *Leishmania* amastigotes inside macrophages. Asterisks indicate parasitophorous vacuoles. Quantification of parasite load observed in macrophages is shown **(E)**. Normalized values represent means ± SEM of 3–4 independent experiments performed in triplicate. (*P < 0.05) compared to the control group (without treatment).

LTB_4_ is essential to resistance to infection by *L. amazonensis*; therefore, it was used as a positive control for parasitic load assay (**Figure 1B**). Cysteinyl leukotrienes LTC_4_ (**Figure 1C**) and LTD_4_ (**Figure 1D**) treatment resulted in fewer *Leishmania* amastigotes in parasitophorous vacuoles than untreated macrophages from BALB/c mice (**Figure 1A**). A similar effect was observed with LTB_4_. The data were quantified (**Figure 1E**), confirming that treatment with Cys-LTs reduces *L. amazonensis* infection by more than 60%.

**Figure 2:**
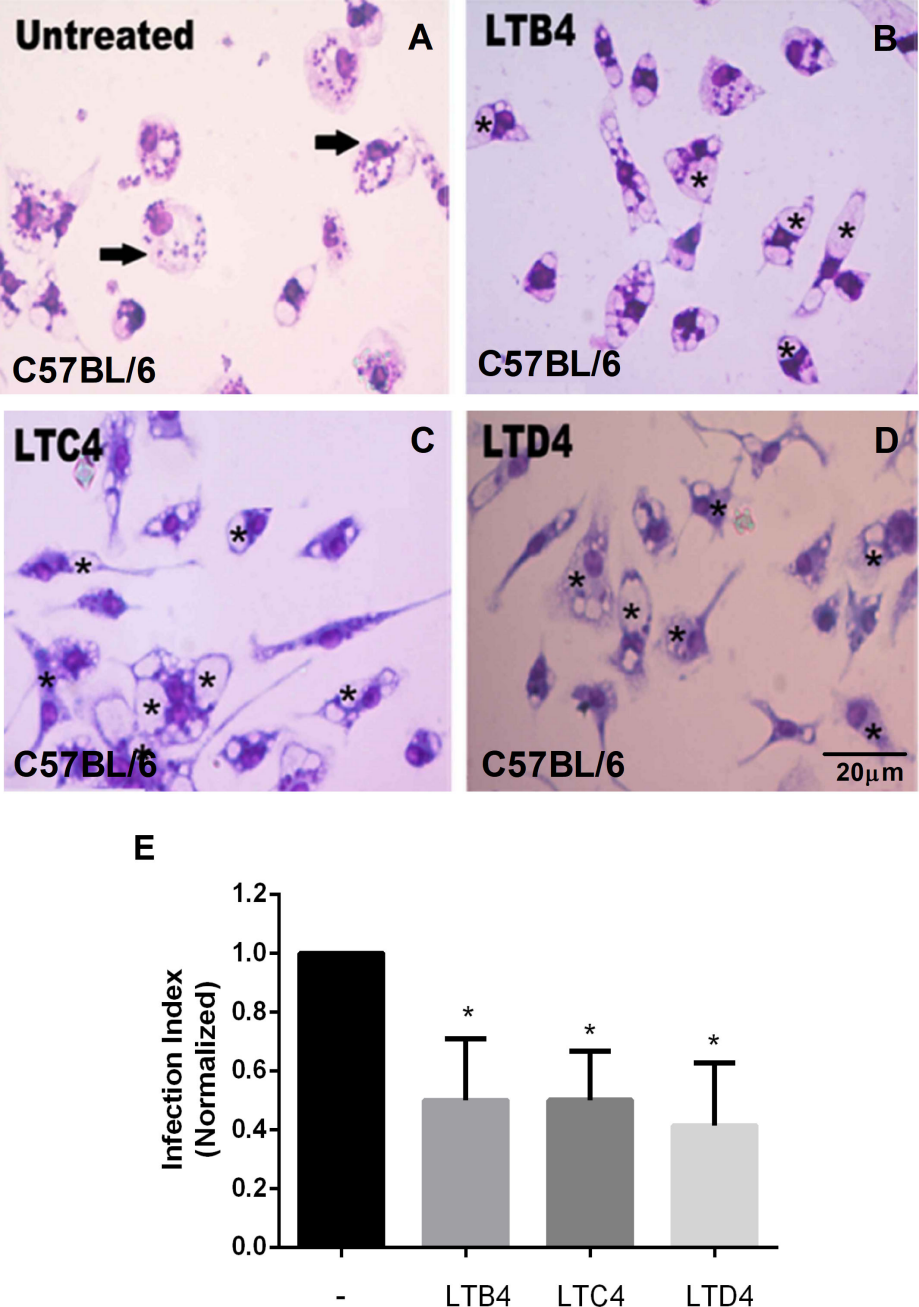
LTs reduce the parasite burden in infected C57BL/6 mouse macrophages. Peritoneal macrophages from C57BL/6 mice were infected with *L. amazonensis* promastigotes at 10:1 (*Leishmania*:macrophage). After 4 h, the free parasites were washed, and after 24 h, infected cells were treated **(B–D)** or not **(A)** with 100 nM of LTB4 **(B)**, LTC_4_ **(C)**, and LTD_4_ **(D)**. Twenty-four hours later, cells were stained with May Grunwald-Giemsa, and the infection index was determined by direct counting under light microscopy. Arrows indicate *Leishmania* amastigotes inside macrophages. Asterisks indicate parasitophorous vacuoles. Quantification of parasite load observed in macrophages is shown **(E)**. Normalized values represent means ± SEM of 3–4 independent experiments performed in triplicate. (*P < 0.05) compared to the control group (without treatment).

As seen with BALB/c macrophages, C57BL/6 peritoneal macrophages treated with LTC_4_ (**Figure 2C**) or LTD_4_ (**Figure 2D**) showed fewer *Leishmania* in the parasitophorous vacuoles (marked with an asterisk) than the untreated group (**Figure 2A**). Similarly, we observed that LTB_4_ treatment reduced the number of *Leishmania* in parasitophorous vacuoles from C57BL/6 macrophages (**Figure 2B**). The data were quantified (**Figure 2E**), and Cys-LTs reduced *L. amazonensis* infection by 50%.

### 3.2 Intralesional administration of cysteinyl leukotriene LTD_4_ reduced cutaneous *Leishmania* lesion progression in footpads

**Figure 3:**
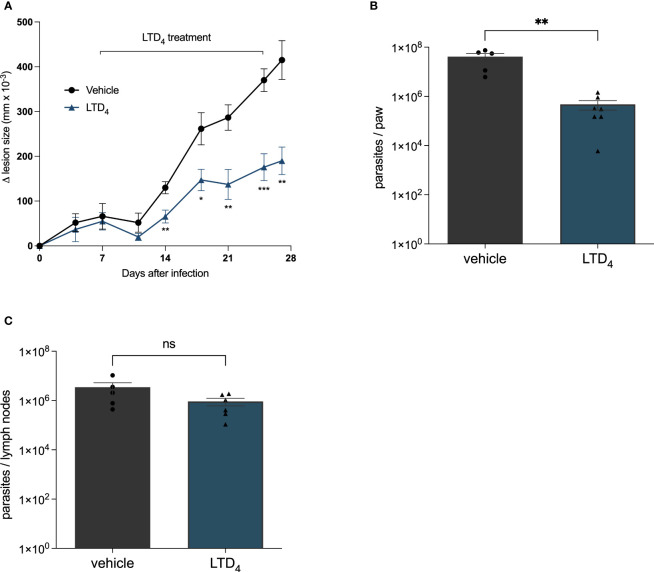
Intralesional treatment with LTD4 induced resistance to L. amazonensis infection in mice. C57BL/6 mice were subcutaneously injected into the footpad with 10^6^ *L. amazonensis* promastigotes. Seven days post-infection (dpi), the mice were treated with 5 ng of LTD_4_ in 20 μL PBS (six doses applied intralesionally twice a week) for 3 weeks (7–27 dpi). **(A)** Lesion size (edema) was determined using a traditional caliper (Mitutoyo^®^) and was expressed as 10^–3^ mm. Animals were euthanized at 29 dpi. The infected paw and popliteal lymph nodes were removed using a limiting dilution assay to measure parasite load in the paw **(B)** and lymph nodes **(C)**. The data represent the mean ± standard error of the mean of the group of animals (n = 5). ***p < 0.001, **p < 0.01 and *p < 0.05 compared to untreated control group. Data were analyzed using Student’s T-test followed by the Mann-Whitney U-test. ns = non-significant.

Next, we analyzed the effect of Cys-LTs in modulating *Leishmania* skin lesions progression in C57BL/6 infected mice. We infected the right footpad with 10^6^ *Leishmania* promastigotes. We administrated cysteinyl leukotriene LTD_4_ intralesionally (twice a week for 3 weeks) seven days after infection. We compared the evolution of lesion size until the twenty-eighth day of infection (**Figure 3**). Lesion size progressed significantly slower in mice treated with LTD_4_ than in those receiving the vehicle solution. From 14 dpi, the treated mice showed significative smaller lesion sizes, and on 28 dpi, lesions in LTD_4_ treated mice were less than half the size of lesions in untreated lesions (**Figure 3A**). LTD_4_ treatment induced a greater than 100-fold reduction in the number of parasites in the paw macerate (**Figure 3B**), while there was no change in the number of *Leishmania* in draining lymph nodes (**Figure 3C**).

### 3.3 ATP-induced Cys-LTs production depends on the P2X7 receptor

ATP is an important signaling molecule in the immune system that binds the P2X7 purinergic receptor (Savio et al., 2018). Our group showed that ATP-treated macrophages control *L. amazonensis* infection (Chaves et al., 2019). LTB_4_ reduces the parasitic load of infected macrophages; additionally, the P2X7 receptor induces phospholipase A2 activation and AA mobilization. We also demonstrated that LTB_4_ production depends on P2X7 receptor activation, as macrophages lacking the P2X7 receptor did not reduce parasite infection or produce LTB_4_ in response to infection.

**Figure 4:**
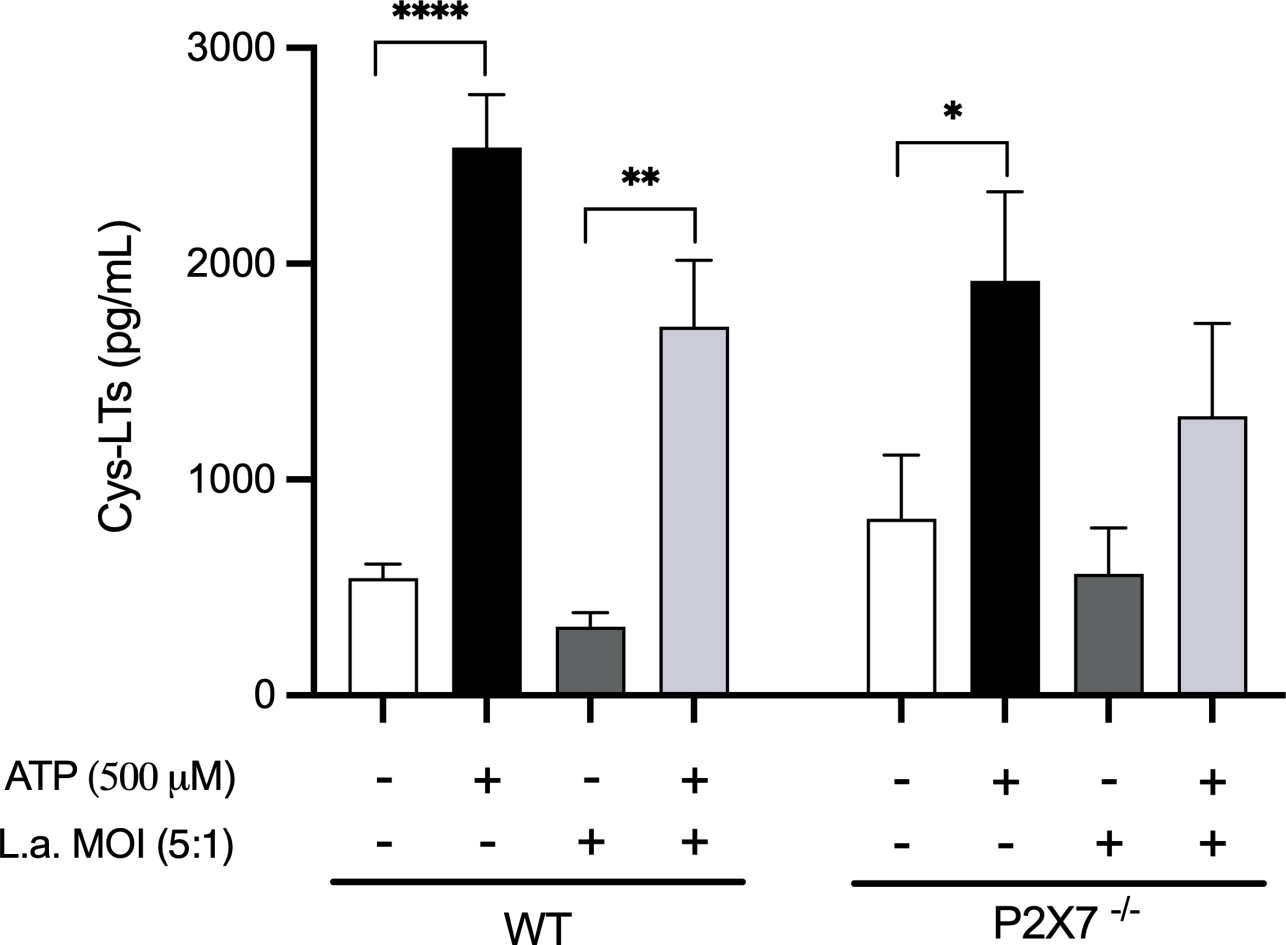
P2X7 receptors activation induces the production of Cys-LTs. Peritoneal macrophages (2 x 10^5^) from wild-type and P2X7^-/-^ mice were plated in 96-well culture plates. After 24 h, the cells were infected with *L. amazonensis* promastigotes at an MOI (5:1) for 4 h. After 24 h of infection, ATP (500 μM) was added to the cultures for 30 min. The supernatants were collected for the measurement of Cys-LTs by EIA. Data are presented as the mean ± standard error of the mean of three independent experiments performed in triplicates. Data were analyzed using analysis of variance followed by Tukey’s Post-test. ****p < 0.0001, **p < 0.001 and *p < 0.05.

Therefore, we investigated whether macrophages would produce Cys-LTs in response to ATP in a P2X7-dependent way. The addition of ATP (500μM) to peritoneal macrophages induced the release of Cys-LTs compared to non-stimulated cells (**Figure 4**). This production appears to be partially dependent on P2X7 receptor as macrophages from P2X7^-/-^ mice produced fewer Cys-LTs in response to ATP. Additionally, *Leishmania* infection decreased the production of Cys-LTs by macrophages, and P2X7-deficient macrophages had their production almost abolished.

## 4 Discussion

LTs, lipid mediators of inflammation, are well-described for participating in the inflammatory processes of chronic, allergic, and autoimmune diseases (Griffiths et al., 1995; Capra et al., 2007; Ohnishi et al., 2008). In this context, Cys-LTs are involved in events such as cell migration and leukocyte adhesion (Dahlén et al., 1981; Yuan et al., 2009; Dannull et al., 2012). In asthmatic patients, these molecules are essential in the respiratory system, participating in goblet cell hyperplasia, vascular permeability, and bronchoconstriction (Manning et al., 1990; Kim et al., 2006; Hashimoto et al., 2009; Kazani et al., 2011). However, their role in antimicrobial defense has been largely overlooked compared to cytokines and chemokines. This discrepancy likely reflects the common view that lipid mediators are exclusively pathogenic and the resultant idea of their pharmacological blockade as the primary objective of pharmacological research (Serezani et al., 2023).

Our group has demonstrated the interplay between the activation of purinergic receptors and LTB_4_ in promoting the resolution of *Leishmania* infection in macrophages (Chaves et al., 2009; Marques-da-Silva et al., 2011; Chaves et al., 2014b; Marques-da-Silva et al., 2018). Our previous study demonstrated that P2X7 receptor activation releases leukotrienes, primarily LTB_4_ (Chaves et al., 2014b). Serezani et al. (2006) demonstrated that LTB_4_ induced the elimination of *L. amazonensis* in infected macrophages *in vitro* and that the 5-LO pathway of arachidonate is necessary for efficient parasite elimination. Cell-specific leukotriene profiles have been described with mast cells and eosinophils synthesizing primarily Cys-LTs, neutrophils and dendritic cells synthesizing primarily LTB_4,_ and macrophages producing a balance of both LT classes (Peters-Golden et al., 2005).

The effect of Cys-LTs in the modulation of intracellular parasite infection is much less clear. The role of Cys-LTs in intracellular parasite elimination was demonstrated almost 30 years ago when Wirth and Kierszenbaum (1985) reported that LTC_4_ facilitated phagocytosis and elimination of *Trypanosoma cruzi* by peritoneal macrophages. The role of Cys-LTs in the modulation of *Leishmania* infection remains undescribed. This phenomenon was also observed for LTB_4_ production by *Leishmania*-infected macrophages, which showed a reduction ability, reinforcing the idea that the parasite impairs LTs production/action to survive within macrophages.

The present study showed that Cys-LTs modulate *Leishmania* infection in peritoneal macrophages from susceptible and resistant mouse strains. The doses of Cys-LTs were based on the literature to activate high- and low-affinity receptors. Adding LTC_4_ or LTD_4_ reduced the parasite index in macrophages, similar to the already described effect of LTB_4_. We detected Cys-LTs production by cultured macrophages responding to extracellular ATP (eATP). Nevertheless, *Leishmania*-infected macrophages appeared to reduce the amount of Cys-LTs produced, suggesting a possible adaptation of the parasite to evade the antiparasitic effect of Cys-LTs.

The P2X7 receptor appears to be partly necessary to Cys-LTs production, as P2X7-deficient macrophages exhibited nearly half the amount of Cys-LTs in response to eATP; *Leishmania* infection prevented Cys-LTs production by macrophages. These *in vitro* results demonstrate that exogenous Cys-LTs can interfere with parasite load in cultured cells, and macrophages can produce Cys-LTs in response to eATP. However, *Leishmania* appears to have developed a way to interfere with Cys-LTs production in infected cells, increasing survival. The need for P2X7 receptor activation by eATP reinforces the putative role of Cys-LTs in synergizing the antimicrobial events in infected macrophages. The mechanisms by which parasite infection decreases LTs synthetic capacity remain to be elucidated. Several possibilities are reasonable, including interference with the enzyme synthesis/activity, receptor expression, or signaling pathways.

Next, we tested the effect of exogenous Cys-LTs on wounds resulting from *L. amazonensis* infection in mice. Our results suggest that exogenous Cys-LTs (specifically LTD_4_) induce a significant reduction in lesion size caused by *Leishmania* infection. The number of parasites in the wound was significantly reduced, suggesting a local effect of Cys-LTs on parasite load. These findings indicate a previously undescribed role of Cys-LT in the leishmanicidal effect in macrophages. Although we used a short-term time of infection (1 week) to accelerate treatment evaluation, all living parasites were likely intracellular and represented active lesions by that time. Future experiments using lesions older than 3–4 weeks will provide insights into leukotriene effects in established infections.

Previous studies showed that inhibiting leukotriene production or its receptor using a 5-lipoxygenase-activating protein inhibitor (MK0591) or LTB_4_ receptor antagonist (U75302) decreased leishmanicidal activity by infected macrophages. However, a CysLT1 receptor antagonist (MK571) did not affect the macrophage infection index (Serezani et al., 2006). These contradictory results indicate the need for further investigation into the molecular mechanisms underlying wound size reduction and the macrophage infection index, both of which were reduced in our experiments.

Cys-LTs bind to CysLT1R and CysLT2R. CysLT1R is the high-affinity receptor, primarily expressed in leukocytes (mostly eosinophils and monocytes/macrophages), basophils, vascular endothelial cells, mast cells, neutrophils, and subsets of B lymphocytes. By contrast, CysLT2R receptors are expressed in the heart, brain, and adrenal glands, despite some overlapping with CyslT1R-expressing cells. Moderate CysLT2R expression was observed in the spleen, lymph nodes, and peripheral blood leukocytes and was more highly expressed in eosinophils (Capra et al., 2007). Nevertheless, there may be an additional Cys-LT receptor because an antagonist of receptors (BAYu9773) failed to inhibit all Cys-LT functional responses (Walch et al., 2002). Therefore, the newly described leishmanicidal effect of Cys-LT may depend on an unknown receptor.

We conclude that the effect of Cys-LTs on *L. amazonensis* infection, either by reducing the parasite index in cultured infected macrophages or hampering skin wound progression in intradermally infected mouse footpads, points to a promising new drug target for CL. Further mechanistic studies should be performed to elucidate its leishmanicidal effects.

## Data availability statement

The raw data supporting the conclusions of this article will be made available by the authors, without undue reservation.

## Ethics statement

The procedures were performed following the guidelines of the Brazilian College of Animal Experimentation (COBEA) and were approved by The Commission for Ethical Use of Research Animals (CEUA) from the Federal University of Rio de Janeiro (UFRJ) n° 077/15 and n° 152/21.

## Author contributions

LN, MM, MT, and LC-S conducted *in vivo* and *in vitro* experiments. AC-J contributed to data analysis and wrote the paper. TR contributed to data analysis. FC-G and BR-B contributed to the *in vivo* experiments. LS contributed to the data analysis and article revision. CC conducted the cysteinyl leukotriene quantification and article revision. RC-S conceived and designed the experiment and revised the manuscript. All authors contributed to the article and approved the submitted version.
